# Evaluation of Cellular Immunity with ASFV Infection by Swine Leukocyte Antigen (SLA)—Peptide Tetramers

**DOI:** 10.3390/v13112264

**Published:** 2021-11-12

**Authors:** Wenqiang Sun, He Zhang, Wenhui Fan, Lihong He, Teng Chen, Xintao Zhou, Yu Qi, Lei Sun, Rongliang Hu, Tingrong Luo, Wenjun Liu, Jing Li

**Affiliations:** 1 State Key Laboratory for Conservation and Utilization of Subtropical Agro-Bioresources & Laboratory of Animal Infectious Diseases, College of Animal Sciences and Veterinary Medicine, Guangxi University, Nanning 530004, China; sunwenqiangsyfq@163.com; 2 CAS Key Laboratory of Pathogenic Microbiology and Immunology, Institute of Microbiology, Chinese Academy of Sciences, Beijing 100101, China; zhanghe901227@126.com (H.Z.); fanwenhui78@aliyun.com (W.F.); helh0728@foxmail.com (L.H.); sunlei362@im.ac.cn (L.S.); 3 Institute of Infectious Diseases, Shenzhen Bay Laboratory, Shenzhen 518000, China; 4 Savaid Medical School, University of Chinese Academy of Sciences, Beijing 100049, China; 5 Institute of Military Veterinary Medicine, Academy of Military Medical Science, Changchun 130122, China; ctcx1991@163.com (T.C.); zhouxtao@Foxmail.com (X.Z.); qiyu0204@sina.com (Y.Q.)

**Keywords:** African swine fever virus, protective antigen, T epitopes, T cell immune response, p72

## Abstract

African swine fever virus (ASFV) causes acute hemorrhagic fever in domestic pigs and wild boars, resulting in incalculable economic losses to the pig industry. As the mechanism of viral infection is not clear, protective antigens have not been discovered or identified. In this study, we determined that the p30, pp62, p72, and CD2v proteins were all involved in the T cell immune response of live pigs infected with ASFV, among which p72 and pp62 proteins were the strongest. Panoramic scanning was performed on T cell epitopes of the p72 protein, and three high-frequency positive epitopes were selected to construct a swine leukocyte antigen (SLA)-tetramer, and ASFV-specific T cells were detected. Subsequently, the specific T cell and humoral immune responses of ASFV-infected pigs and surviving pigs were compared. The results demonstrate that the specific T cellular immunity responses gradually increased during the infection and were higher than that in the surviving pigs in the late stages of infection. The same trend was observed in specific humoral immune responses, which were highest in surviving pigs. In general, our study provides key information for the exploration of ASFV-specific immune responses and the development of an ASFV vaccine.

## 1. Introduction

African swine fever virus (ASFV) infects domestic pigs, leading to acute hemorrhagic disease, which causes huge economic losses to the pig industry. Since its initial outbreak, the virus has rapidly spread to countries in Europe and Russia and recently to Belgium, China, Vietnam, and beyond [1,2]. Due to the lack of an effective vaccine, limited measures to prevent and control the virus include the isolation of infected farms and culling of infected pigs. The virus that causes African swine fever belongs to the *Asfarviridae* family, a large icosahedral double-stranded DNA virus with a linear genome of 189 kb, containing more than 180 genes [3]. It circulates in its natural hosts (warthogs, bush hogs, and soft ticks), which show no signs of disease, while ASFV has caused explosive deaths when infecting domestic herds [4]. ASFV has many coding proteins, and no single antigen has been found to provide complete protection. The current progress of ASFV vaccine research shows that an attenuated live vaccine can produce considerable protective effects, but there are biosafety risks [5,6,7,8]. Inactivated vaccines, subunits, and vector vaccines provide limited or no protection, and the development of novel multicomponent antigenic vaccines against viruses remains experimental [9,10,11,12].

The failure and slowness of ASFV vaccine development is due to the fact that the infection process of the virus is not well defined, and key protective antigens have not yet been identified. It is well known that the entry of viruses into host cells is receptor-mediated, and the main targets of replication are monocytes and macrophages [13,14,15]. However, the exact endocytosis mechanism remains unclear. Previous studies demonstrated that cytokine storms occur after ASFV infection [16,17]. Furthermore, the cellular immune response after ASFV infection has also been studied. Previous studies reported that IL-2 and IFN-γ are found after the peripheral blood mononuclear cells (PBMCs) of surviving pigs are stimulated by the virus. Furthermore, impaired T cell responses are noted in domestic pigs and wild boars upon infection with a highly virulent ASFV strain [18]. Importantly, an anti-CD8 antibody can abolish the protection of immunized pigs against highly virulent ASFV [19].

An unusual aspect of the pig immune system is the large number of CD4+CD8+ double-positive (DP) T cells [20]. After a virulent ASFV infection, the frequency of DP T cells in the peripheral blood and lymphatic organs of domestic pigs increases, while the frequency of CD4+ T cells decreases, but there is no proliferative activity or proper perforin expression. Notably, the perforin expression in DP T cells is lost at 5 dpi but recovers after two days. The DP T cells of wild boars have high proliferative activity. However, there are no detailed data on the role of DP T cells in ASFV infection [21]. Regardless, there is considerable evidence that CD8+ T cells play a key role in protection.

However, the changes in specific humoral and cellular immunity in pigs infected with ASFV remain unclear. In this study, the PBMCs of ASFV survivor pigs were used to screen for the antigens of the main activated T cells. Furthermore, T cell epitopes of key proteins were predicted, and positive T cell epitopes were screened. Finally, swine leukocyte antigen (SLA) tetramers were prepared from positive T cell epitopes to evaluate specific cell immunity and humoral immunity in infected and surviving pigs. Based on this investigation, we illustrate the key protein profiles specific to the virus and reveal the role and mechanism of the host viral T cell response to ASFV infection. The SLA tetramers developed in this study provide an effective tool for the evaluation of T cell immunity after ASFV infection and vaccine immunization.

## 2. Methods and Materials

### 2.1. Ethics Statement

The pig experiments involved in the study were conducted in a BSL-3 level laboratory in the Institute of Military Veterinary Medicine, Academy of Military Medical Science, and were approved by the ethics committee. The analytical samples and protocols used in this study were approved by the Institute of Microbiology, Chinese Academy of Sciences, Research Ethics Committee (license number: PZIMCAS2019003; Permission date: 1 March 2019).

### 2.2. Cells and Viruses

A highly virulent field strain of ASFV (ASFV-SY18, GenBank accession number MH766894) isolated in China was used. Samples were collected for African swine fever testing and surveillance under the agreement between the Ministry of Agriculture and Rural Affairs of the Chinese Government and farm owners of the Centre for Animal Disease Control and Prevention of Guangdong Province, with the protocols established by the World Organization for Animal Health. Virus stocks were propagated in primary porcine alveolar macrophages (PAMs) and stored at −80 °C at the Institute of Military Veterinary Medicine, Academy of Military Medical Science. PAMs were collected from 35-day-old specific-pathogen-free (SPF) pigs and then grown in an RPMI 1640 medium (Thermo Scientific, Waltham, MA, USA) supplemented with antibiotics–antimycotics (10,000 IU/mL of penicillin, 100 mg/mL of streptomycin, and 0.25 mg/mL of Fungizone) and 10% heat inactivated fetal bovine serum (FBS, Hyclone, Logan, UT, USA) at 37 °C with 5% CO_2_ according to established procedures. The viral titer was determined based on macrophage cultures (TCID_50_/mL). PBMCs and serum samples of surviving pigs (n = 20) were collected from ASFV-infected pigs that have survived in animal infection experiments. Anticoagulant blood was collected through the anterior vena cava and PBMCs were isolated from whole blood using Histopaque-1077 density gradient solution (Sigma-Aldrich, Burlington, MA, USA).

### 2.3. Construction of Protein Expression Vectors

Full-length genes encoding the protein sequences of ASFV-SY18 p72-B646L, pp62-CP530R, CD2v-EP402R, and p30-CP204L were synthesized (GenScript, Nanjing, China) with codon optimization and cloned into the prokaryotic expression vector pET-32a. The construction of SLA-peptide tetramer (Tetra-P334, Tetra-P351, and Tetra-P366)—peptides, β2M, SLA-I extracellular domain, and a 15-amino-acid acceptor peptide (also known as the Avi-tag) were fused in tandem by a flexible linker. The genes were synthesized and cloned into the prokaryotic expression vector pET-22. All of the above proteins carried a C-terminal His tag.

### 2.4. Expression and Purification of Protein

*E. coli* BL21 (DE3) cells were transformed with the recombinant expression plasmid, and a single colony was cultured in an LB medium overnight at 37 °C. Then, this culture was used to inoculate a large culture flask at a 1:100 ratio. IPTG (final concentration 1 mM) induction was performed for 6 h until the optical density (OD) value reached 0.5. Cells were collected by centrifugation at 2200× *g* for 10 min and resuspended in PBS for ultrasonic lysing. The inclusion bodies and supernatant were separated by centrifugation at 2200× *g* for 30 min, and the inclusion bodies were washed twice with a wash buffer (0.5% Triton-100, 50 mM Tris pH 8.0, 300 mM NaCl, 10 mM EDTA, and 10 mM DTT). Finally, 8 M Urea was used to dissolve the inclusion bodies. After purification through a HisTrap FF crude column, the protein was added dropwise to a refold buffer (100 mM Tris-HCl pH 8.0, 400 mM L-arginine, 2 mM Na-EDTA, 5 mM glutathione, and 0.5 mM oxidized glutathione) and stirred for 24 h at 4 °C. Three additional SLA-peptide tetramers (Tetra-P334, Tetra-P351, and Tetra-P366) were placed into a biotin buffer (20 mM Tris and 150 mM NaCl), labeled with biotin using a biotinylation kit (BirA-500, Avidity, VWR International, LLC., Radnor, PA, USA), and then size exclusion chromatography (GE, Boston, MA, USA) was used to isolate unbound free biotin and biotin protein. Purity was assessed by SDS-PAGE, and the concentrations of the proteins were determined using a BCA protein assay kit (CW Bio, Cambridge, MA, USA). Recombinant protein was stored at −80 °C.

### 2.5. Porcine IFN-γ ELISPOT Assay

To detect specific T lymphocyte responses, IFN-γ ELISPOT assays were performed by pig IFN-γ single-color ELISPOT kit (pIFNgp-1m/10, CTL). The PBMCs of surviving pigs (n = 20) were isolated under aseptic conditions and stored in liquid nitrogen. PBMCs were thawed and resuspended in a 1640 medium. The PBMCs suspensions (4 × 10^5^/well), p72, pp62, p30, CD2v, and inactivated virus diluents (10 μg/mL) were added to 96-well plates precoated with IFN-γ antibodies. ConA was added as a positive control. Cells incubated without stimulation were employed as the negative control. After 18 h of incubation, the biotinylated antibody, streptavidin-HRP, and fresh substrate were added to the plates. Finally, the reaction was stopped by rinsing the plate with deionized water. The same method was used to detect peptides ELISPOT assays, and PBMCs samples from 16 of 20 surviving pigs were selected. The number of spots was determined using a CTL Spot Reader (CTL, New York, NY, USA).

### 2.6. Immune Epitope Database (IEDB) Search and Peptide Identification

The Immune Epitope Database (IEDB, iedb.org (accessed on 1 May 2019) is a companion website to the IEDB that provides computational tools focused on the prediction and analysis of B and T cell epitopes, including antibody, T cell, and MHC binding contexts associated with infectious, allergic, autoimmune, and transplant-related diseases. The predicted peptide affinity is expressed by percentile rank, and other parameters are expressed by relative risk, mean difference, and their 95% confidence intervals (threshold value ≤1). We used *p*-scores to rank the best treatments per peptide. The peptide was synthesized by the Nanjing GenScript Company (purity >95%). The affinity of the peptide to the SLA-I allele was predicted using IEBD NetMHCpan EL 4.1 (95% confidence intervals), and the allele with the highest score and the corresponding peptide was selected to prepare the SLA-peptide tetramer.

GalaxyPepDock protein–peptide docking software was used to enable the molecular docking between alleles and peptides, and the prediction results of peptides and SLA-I crystal complexes were obtained. The docking accuracy is expressed by the prediction accuracy, which was estimated by linear regression of the results of the PeptiDB test set and by a linear model that correctly predicted the proportion of binding site residues and temple-target similarity (measured by protein TMscore and SInter).

### 2.7. Animal Experiments

The surviving pig samples were collected from pigs that had survived exposure to ASFV in previous animal experiments and were infected with ASFV through direct contact with artificially infected pigs. PBMCs samples were frozen in liquid nitrogen and serum was stored at −80 °C (Supplementary Table S1).

A total of 14 50-day-old Duroc × Large White × Landrace crossbred female SPF pigs weighing of 19–21 kg were used in the experiment. The SPF pigs were screened by conventional or qPCR assays before the experiment. In the infection group, 1 pig (a#) was intramuscularly inoculated at the left hind limb with 1 mL of PBS-diluted 10^3^ TCID_50_ virus and cohabitated with other uninfected pigs (b# to g#) to mimic the natural contact route of infection. A pig (h#) was intramuscularly injected with PBS and cohabitated with other pigs (i# to n#) as a placebo group. Rectal temperature was measured every 5 days at 10 am, and PBMCs and serum were collected on days 0, 10, and 20 post infection (dpi). The PBMCs and serum samples of s6 surviving pigs (Pig 2#, Pig 3#, Pig 5#, Pig 7#, Pig 10#, and Pig 14#) were selected as convalescent pigs. Euthanasia was performed at the end of the experiment or when the prognosis was poor. In challenge animal experiments, the clinical statuses and food intakes of the pigs were monitored and recorded at 9 am daily.

### 2.8. ELISA

ELISA plates (96-well) were precoated with the p72, pp62, and p30 proteins (ID Screen African Swine Fever Indirect, ID.vet). Serum was diluted 2-fold and added to each well, and the plates were incubated with goat anti-pig IgG-HRP and developed by the addition of 100 µL of 3,3′,5′,5-tetramethylbenzidine (TMB). Finally, 100 µL of 2 mmol/L H_2_SO_4_ was added to terminate the reaction, and then the light absorption of the plate was measured at 450 nm using a microplate reader (Thermo Scientific, Waltham, MA, USA). The OD value of the highest dilution was 2.1-fold higher than that of the negative control at the same dilution and was used as the serum endpoint dilution titer. Each experiment was performed 3 times.

### 2.9. Fluorescence-Activated Cell Sorting (FACS)

Peptide-specific CD8+ T lymphocyte immune responses were characterized by FACS assays. In brief, PBMCs were isolated from anticoagulant blood and were stained with CD8α-FITC (Clone: 76-2-11, BD, East Rutherford, NJ, USA) surface marker and tri-tetramer-PE mixture (Tetra-334-PE, Tetra-351-PE and Tetra-366-PE). The cells were then resuspended in the FACS buffer. All labeled lymphocytes were analyzed on an FACSCaliber flow cytometer (BD Biosciences, San Jose, CA, USA), and the data were analyzed using FlowJo V10 version.

## 3. Results

### 3.1. Surviving Pigs Infected with ASFV Produced Protein-Specific T Cells Targeting p30, pp62, p72, and CD2v Proteins

The genes encoding ASFV proteins (p30, pp62, p72, and CD2v) were cloned into the pET-32a vector after codon optimization and were expressed in *E. coli* BL21 (DE3) cells. All of the proteins were expressed in inclusion bodies (Supplementary Figure S1A–D), which were dissolved in 8 M urea. The inclusion bodies were purified by Ni-affinity chromatography using the AKT protein purification system. SDS-PAGE shows that all four ASFV proteins were purified with expected molecular weights (Supplementary Figure S1E–H). It is worth mentioning that the expression of CD2v protein was low, but after enrichment and purification, the protein amount required for experimentation was achieved (Supplementary Figure S1). The purified proteins were refolded, dialyzed, underwent endotoxin removal, were quantified, and then stored at −80 °C for future use.

**Figure 1:**
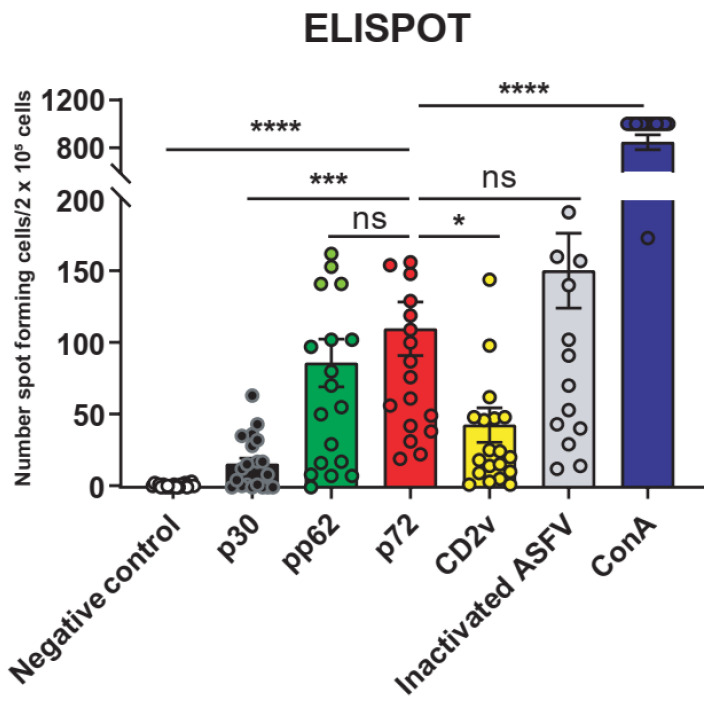
Peripheral blood mononuclear cells (PBMCs) from surviving ASFV-infected pigs responded to ASFV protein expression. The ELISPOT assay verified the ability of the 4 ASFV proteins and the inactivated ASFV to stimulate the production of IFN-γ on PBMCs of the surviving pigs (n = 20). A total of 0.5 μg/mL concanavalin A (Con A) was used as a positive stimulation. The data are shown as the mean ± SEM. *p*-values were determined by one-way ANOVA (ns, *p* > 0.05; * *p* < 0.05; *** *p* < 0.001; and **** *p* < 0.0001).

ELISPOT assay results show that PBMCs of ASFV-infected surviving pigs (n = 20) produced IFN-γ after stimulation by p30, pp62, p72, and CD2v protein and the inactivated virus, and ConA was used as a positive control for stimulation (Supplementary Figure S2). Among these factors, pp62 and p72 proteins and the inactivated virus showed the strongest stimulating ability. Furthermore, p72 protein stimulated PBMCs to produce the most spots among the four proteins (Figure 1). The reason for this may be that p72 protein is a capsid protein with a large content, which is degraded by macrophages or dendritic cells (DCs) into a polypeptide that is presented by SLA-I and plays an important role in cellular immunity. Therefore, p72 protein is the preferred target protein for screening T cell epitopes of ASFV.

### 3.2. Prediction of the SLA-I-Restricted T Cell Epitopes of the p72 Protein

**Figure 2:**
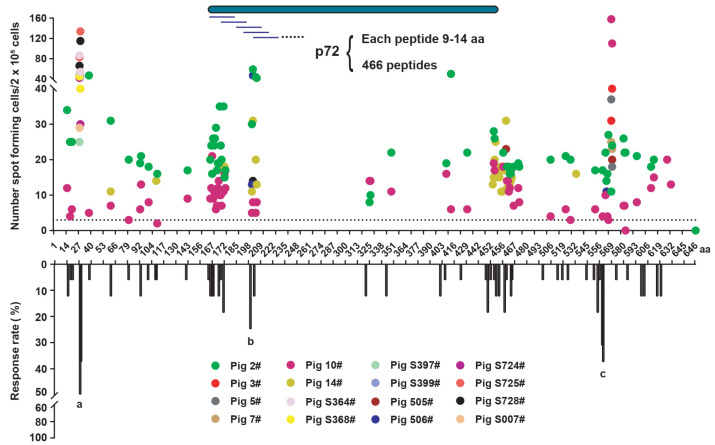
Prediction of p72 porcine leukocyte antigen (SLA) class I-restricted T cell epitopes and screening of positive epitopes. The p72 protein (full length of 646 aa) contains 466 possible peptides, each about 9–14 aa (Supplementary data). The screening of positive epitopes through the peptide library for stimulation of the PBMCs of surviving pigs (n = 16) by ELISPOT assay. Response rates of various peptides in surviving pigs (n = 16). (a) Peptide 351 (P351); (b) peptide 334 (P334); and (c), peptide 366 (P366).

A total of 466 peptides were screened using IEDB MHC-I Binding Predictions. Each peptide contained 9–14 amino acids. ELISPOT assays demonstrated that full-length p72 protein was distributed with SLA-I-restricted T cell epitopes, and most of the positive epitopes were clustered at the N-terminus (1–209) and C-terminus (442–619), and interspersed with other regions. Different pigs have different SLA-I alleles, so the peptides presented may be different. Among them, peptide 351 (P351), peptide 334 (P334), and peptide 366 (P366) had higher response rates in the surviving pigs (n = 16), as shown in a, b, and c in Figure 2. To obtain a broad spectrum of peptides, we evaluated the dynamics of peptide-specific T cell immunity after ASFV infection using the MHC-tetramer technique. Unfortunately, 100% of the response T epitopes were not filtered. Therefore, to ensure the coverage of most pigs, we used all three peptides screened for preparation of SLA-I tetramers.

### 3.3. To Predict the Affinity of Peptides with SLA-I Alleles and Construction of SLA-Peptide Tetramers

**Figure 3:**
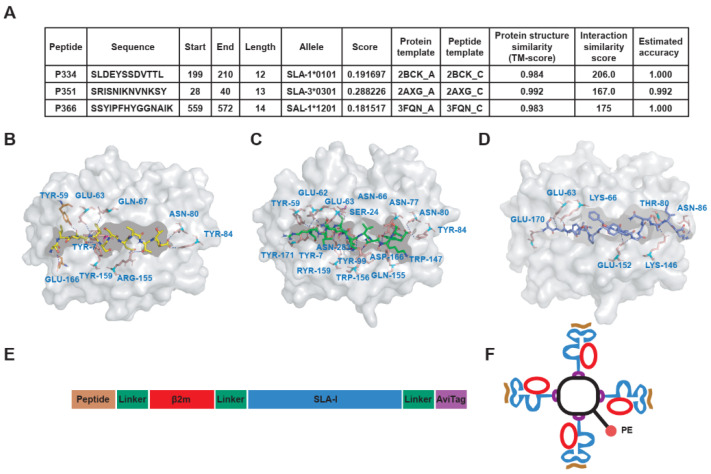
Prediction of SLA-I allele affinity of positive peptides, complex crystal structure analysis, and construction of SLA-I tetramers. (**A**) The affinity of peptides (P334, P351, and P366) and SLA-I alleles was predicted by using IEDB MHC-I Binding Predictions software. The best matching alleles with the highest score were selected. The higher the score is, the better the binding. (**B**–**D**) The crystal structures of the interaction complex between peptide and SLA-I allele were predicted by GalaxyPepDock protein peptide docking software, and the structure model with the highest estimated accuracy value was selected as the optimal structure of the complex. The crystal structure shows the interaction between amino acids and peptides in the SLA-I binding pocket. P334 interacts with SLA-I*0101 (**B**), P351 interacts with SLA-I*0301 (**C**), and P366 interacts with SLA-I*1201 (**D**). (**E,F**) Schematic diagram of SLA-tetramer construction. The single-chain trimer (SCT) technique was used to connect the peptide, light chain β2m, and heavy chain SLA-I in series with flexible linkers (**F**). The monomeric SCT was assembled into tetramers by streptavidin with PE fluorescence.

The results reveal that P334 has the highest affinity with allele SLA-1*0101, P351 has the highest affinity with allele SLA-3*0301, and P366 has the highest affinity with allele SLA-1*1201. In addition, the crystal structure of peptides and allele complexes was predicted with the highest accuracy (Figure 3A). The predicted crystal structure of the SLA-peptide complex shows that the peptide interacts with the amino acids in the SLA pocket to form the complex. In the P334 peptide, the amino acids SDYTL are the core epitope sequence; in the P351 peptide, SRISNIKNNKY is the core epitope sequence; and in the P366 peptide, SSYGGAK is the core epitope sequence (Figure 3B–D). The residues interacting with epitope amino acids and allele pockets are shown in Supplementary Figure S3. Strictly speaking, there is still a deviation between the results predicted by the software and the actual results. Overall, the prediction results indicate that these peptides are most likely to be presented by the corresponding alleles, providing a reference for the next step in the construction of tetramers.

The construction of SLA-tetramers adopted single-chain trimer (SCT) technology to connect the peptide, light chain β2m, and heavy chain SLA-I in a series with flexible linkers (Figure 3E). Four-molecule SCT formed SLA tetramers with fluorescence by cross-linking with one molecule of streptavidin (Figure 3F). SCT proteins were expressed in inclusion bodies and purified by Ni-affinity chromatography (Supplementary Figure S4A–F). The purified SCT proteins were refolded, dialyzed, concentrated, and then biotinylated. Biotinylated protein and free biotin were separated by size exclusion chromatography (Supplementary Figure S4G–I). The biotinylation efficiency of SCT was evaluated by SDS-PAGE gel-shift assays. We found that it was sufficient for further experiments, and there were trace monomers of SCT-P334, SCT-P351, and SCT-P366, as well as various forms of aggregates (Supplementary Figure S5).

### 3.4. The Level of Humoral Immunity Peaked during the Recovery Period of ASFV Infection

**Figure 4:**
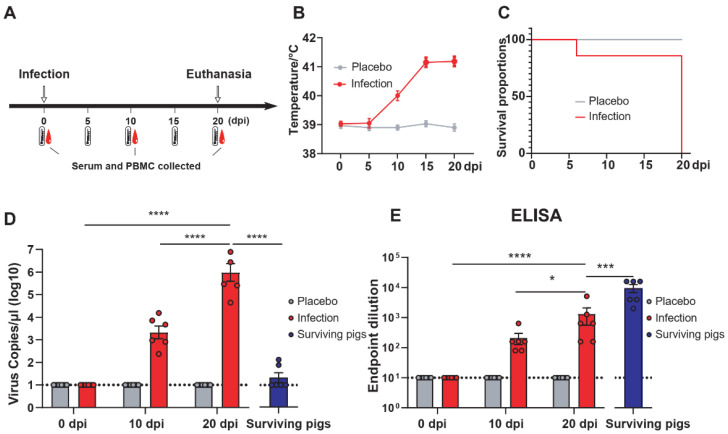
Clinical trials and humoral immunity levels of ASFV-infected pigs and the comparison with surviving pigs. (**A**) Procedures for ASFV infection in pigs. In the infected group, a pig (a#) was infected with ASFV by intramuscular injection and cohabitated with other pigs (b# to g#). In the placebo group, a pig (h#) was intramuscularly injected with PBS and cohabitated with other pigs (i# to n#). The PBMCs and serum samples of surviving pigs (Pig 2#, Pig 3#, Pig 5#, Pig 7#, Pig 10#, and Pig 14#) were selected as convalescent pigs. Rectal temperature monitoring and blood collection was performed at the indicated time points (dpi) after infection. (**B**) Trend of rectal temperature at different time points in ASFV-infected pigs. (**C**) Survival curves of pigs challenged with ASFV (1 artificial infection and the other six contact infection pigs) or PBS (one intramuscularly injected with PBS and the other 6 contact-infected pigs) (**D**) Blood viral load of ASFV-infected pigs at different time points. (**E**) Titers of anti-p72, -pp62, and -p30 specific IgG in pigs infected with ASFV. The dotted horizontal lines represent the quantitative limits of ELISA and viral RNA load. The data are shown as the mean ± SEM. *p*-values were determined by one-way ANOVA (* *p* < 0.05; *** *p* < 0.001; and **** *p* < 0.0001).

The serum and PBMCs of pigs were collected, and the pig’s rectal temperature was measured according to the indicated time point (Figure 4A). According to the clinical rectal temperature index, some pigs developed a fever five days after infection. The infection rate was relatively low because of natural contact. The rectal temperature peaked at 15 dpi and remained unchanged at 20 dpi (Figure 4B). This artificially infected pig (a#) was euthanized at 6 dpi due to acute infection. The other pigs survived during the observation period with poor prognosis and were euthanized 20 dpi later (Figure 4C). Viral load and ELISA results were consistent with rectal temperature. The difference was that the viral load in the serum of the surviving pigs during the recovery period was at a low level, but the detection of viral DNA indicated that the surviving pigs were still infectious (Figure 4D). The specific IgG antibody titers of p30, pp62, and p72 proteins were detected by ELISA. We found that the antibody level of the surviving pigs reached the highest level. Interestingly, the pig antibody level during the infection period also reached a high level, but the clinical signs were still obvious (Figure 4E). It may be that the protective efficacy of antibodies is limited. In general, the level of humoral immunity continued to rise after ASFV infection, reaching a peak during the recovery period.

### 3.5. The Specific T Cell Immune Response Was Continuously Enhanced and Peaked at the Later Stage of Infection

The T cell immune response after ASFV infection was detected by flow cytometry with SLA-tetramers. In previous work, we constructed three SLA-tetramers, and functional verification tests were performed by staining the PBMCs of surviving pigs with SLA-tetramers, monitored via flow cytometry. The gating strategy for PBMCs staining flow cytometry is shown in Supplementary Figure S6. The results show that staining by the three mixed SLA-tetramers was superior to that of each alone, which may be related to the SLA-I genotype of the pigs, so the mixed SLA-tetramers were used in the subsequent experiments (Supplementary Figure S7).

**Figure 5:**
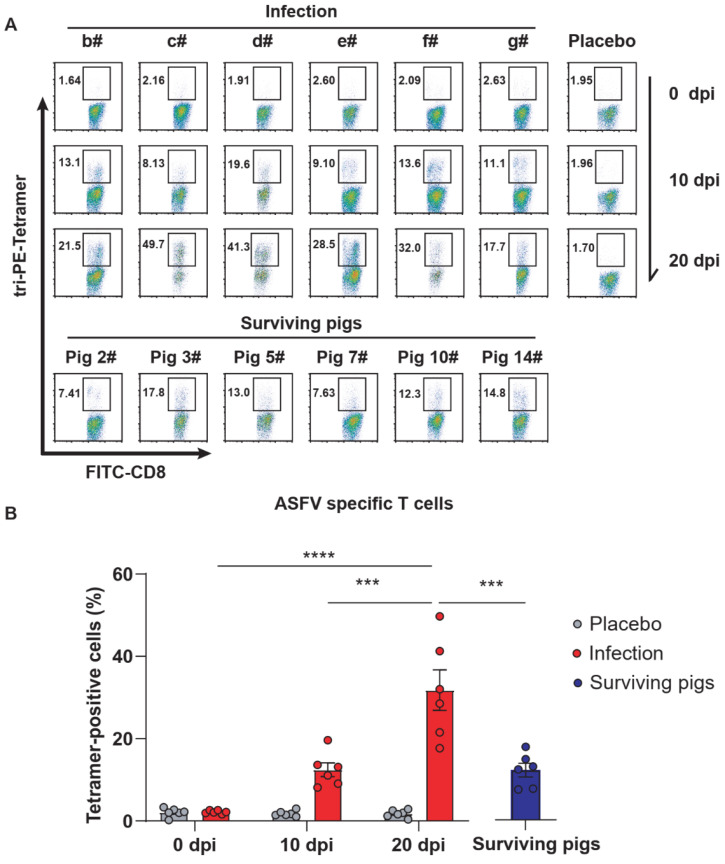
Differences of CD8+ T cells between ASFV-infected pigs and surviving pigs at different time points. (**A**) The proportion of CD8+-specific T cells after ASFV infection was measured by flow cytometry assays of PBMCs stained with tri-PE-tetramer (the mixture of PE-Tera-P334, PE-Tera-P351, and PE-Tera-P366). (**B**) Statistical analysis of the proportion of CD8+-specific T cells after ASFV infection at different time points. The data are shown as the mean ± SEM. *p*-values were determined by one-way ANOVA (*** *p* < 0.001; and **** *p* < 0.0001).

After ASFV infection, the specific T cell immune response continued to increase, reaching the highest peak in the later stage of infection. In contrast, the specific T cell immune response declined during the recovery period (Figure 5A,B). This is because the cellular immune response was suppressed after the virus was removed. Although the T cell response reached a very high level, the condition of the infected pigs showed no signs of improvement, and only a few pigs survived. Overall, the specific T cell immune response was rapidly activated after ASFV infection and gradually decreased during the recovery period.

## 4. Discussion

ASFV infection can cause acute hemorrhagic fever in domestic pigs and wild boars. The mechanism of infection and key protective antigens are not entirely known, hampering vaccine research. Most studies indicate that CD8+ T cells play a vital role in providing protection [19,22,23,24]. Live attenuated vaccines can provide strong protection to pigs, possibly due to the stimulated T cell immunity response [6,8,25]. In our study, we identified key antigens that stimulate T cell immunity in surviving pigs and explained differences in specific cellular and humoral immunity between the acute phase of ASFV infection and the convalescent phase of viral infection.

Most reports confirm that T cell immunity plays an important role in the host immune response, so it was important to screen for CD8+ T cell epitopes of ASFV to elucidate the T cell immunity response of the host against the virus [6,26,27]. The T cell epitopes of CD2v and C-type lectin were scanned through the PBMCs of immunized pigs. The results demonstrated that the positive peptides were found in two proteins and presented agglomerative distribution on the proteins [28]. In another study, 3818 peptides in 165 pools corresponding to 133 open reading frames were selected to screen positive T cell peptides against ASFV immune lymphocytes by IFN-γ ELISPOT assays [29]. The peptide pools of p30, pp62, and p72 that induced significant IFN-γ responses in ASFV-immunized porcine lymphocytes were consistent with our results. In addition, in our study, we found that the two ends of p72 can better induce the production of IFN-γ than truncated-p72, which is also consistent with our peptide results. In contrast, p72 was much weaker than pp62 and p30 in inducing IFN-γ production. This may be the difference between T cellular immunity in surviving pigs and ASFV-immunized pigs [29]. Importantly, different pigs recognize different peptides, and this phenomenon was also found in this study. This is due to the different SLA genotype of different pigs, so the pig breed should be considered when screening for peptides. In general, our study emphasized the importance of p72 in stimulating cellular immunity responses and provides a comprehensive screening of the T epitope of the p72 protein.

The major focus of our study was to characterize the specific T cell immunity and humoral immunity responses in ASFV-infected pigs compared to surviving pigs. Previous studies reported that IL-2 and IFN-γ are produced after the PBMCs of surviving pigs are cultured with virus or virus-infected cells in vitro [30], which was also confirmed in our study. Furthermore, CD8+ lymphocytes depletion assays indicated that CD8+ lymphocytes play an important role in the protective immune response to ASFV infection and that anti-ASFV antibody alone was not sufficient to protect pigs from virulent OUR/T88/1 challenge. Furthermore, Alonso et al. demonstrated that only purified CD8+ lymphocytes—but not purified CD4+ lymphocytes—have CTL activity, and this activity is blocked by anti-CD8+ antibody, though nearly 20% of the CD8+ lymphocytes co-expressed CD4+ [30]. Importantly, this DP T cell population was highlighted in different studies. Jane et al. found that during the infection period, the CD8+ T cells in the blood decreased, while the proportion of DP cells increased in domestic pigs [18]. Early studies on virus-specific immune responses in DP cells show that the mode of action is MHC class II restricted, with T-helper cell functions, and that CD4+, rather than CD8+, is the key molecule involved. In other words, DP cells express their MHC class II restriction through CD4+ molecules, which is the same as CD4+ cells [31,32]. Whether the DP cell population is involved in cytotoxic activity of ASFV needs further exploration. Our results make it clear that the proportion of CD8α+ tetramer+ double positive T cells continues to rise during infection, proving that the specific CD8+ T cell immune response has been activated during ASFV infection. A similar phenomenon was found when CD8β+ perforin+ double positive T cells increased when non-immune and immunized pigs were challenged with virulent infection [21].

Another concern is that the immune responses to artificial and contact infections may be different, though death is the result for both types of pigs. It is worth considering that the cellular immune response may be activated, but death still occurs. There may be other reasons as well. It is well known that most acute infection deaths are caused by storms of inflammatory factors [33,34,35,36,37]. Inflammatory cytokine storms can cause a variety of diseases, including infection, acute respiratory distress syndrome, and sepsis. Importantly, storms of inflammatory cytokines caused by ASFV have been reported [16]. Assuming that cytokines produced after ASFV infection are controlled, infected pigs may survive. As in the early days of the COVID-19 epidemic, treatment options are an effective means of controlling the inflammatory cytokine storm in the absence of specific drugs [38,39,40,41]. In future studies, attention should be paid to these aspects of inflammation caused by ASFV. Taken together, our results characterize differences in specific cellular and humoral immune responses in infected pigs compared to surviving pigs. These data will provide information for future research on ASFV-specific immunity and vaccine development.

In vaccine development, it is important to find the key protective antigen. Pigs immunized with p30, p54, p72, CD2v, or an antigen library had no or partial protection against acute ASFV. When ubiquitin was expressed in-frame with these viral genes, partial protection was obtained, corresponding to a robust CD8+ T cell response, in the absence of specific antibodies [9,10,11,42,43,44]. A recent approach using a combination of selected virus-specific proteins and cDNAs was able to induce a robust immune response in terms of in vitro neutralizing antibodies and IFN-γ production in vivo. However, vaccinated pigs were not protected against virulent challenge with the Armenia07 strain [12,45]. As for the development of a viral vector vaccine, similar to the subunit vaccine, the vaccine can induce a specific immune response but only low or partial protection [27,29,46,47]. Previous studies have sought to find protective antigens and have not shown in detail which antigens play a role in producing protective effects. In this study, our results demonstrate that different antigens stimulated different levels of the T cell immune response in surviving pigs. All four proteins can stimulate the production of IFN-γ in the PBMCs of surviving pigs, among which p72 and pp62 are the most intense. This method can be used as a reference for large-scale screening of antigens that can stimulate the T cell immune response and provide protection for surviving pigs.

The results of this study provide reliable data and support for studying immune response after ASFV infection. The SLA-peptide tetramers were used to analyze the specific cell immunity of infected pigs. The immune response to ASFV infection provides the basis for studying the invasion mechanism, vaccine target antigens, and prevention and control strategies for ASFV infection. At the same time, these results raise many follow-up scientific questions for further research. For example, there are cases where the cellular immune response has been activated, but death still occurs. Thus, the mechanism of infection remains to be further explored. In addition, an ASFV live attenuated vaccine provides protection against the virus strain, which requires further study of properties such as specific cellular immune responses in immunized pigs.

## Data Availability

Further information and requests for resources and reagents should be directed to J.L. or W.L.

## Supplementary Materials

The following are available online at https://www.mdpi.com/article/10.3390/v13112264/s1, Figure S1: The expression and purification of ASFV proteins in E. coli BL21 was detected by SDS-PAGE, Figure S2: The ELISPOT assays of protein-specific T cells, Figure S3: Each amino acid of the epitopes P334, P352, and P366 interact with amino acid in the SLA-I allele pocket, Figure S4: The expression and purification of tetramer protein was detected by SDS-PAGE, Figure S5: The biotinylation efficiency of the SLA-I-peptide complex was evaluated by SDS-PAGE gel-shift assays, Figure S6: Gating strategy for PBMCs staining flow cytometry, Figure S7: The proportion of CD8+ specific T cells of surviving Pig 10# was measured by flow cytometry assays of PBMCs stained with PE-Tera-P334, PE-Tera-P351, PE-Tera-P366 and tir-PE-Tetramer, Table S1: The general information of the surviving pigs.
