# Supplementary material for: Evaluation of Cellular Immunity with ASFV Infection by Swine Leukocyte Antigen (SLA)—Peptide Tetramers
Source: Viruses. 2021 Nov 12;13(11):2264. doi: 10.3390/v13112264 (PMC8617699; doi:10.3390/v13112264)
Supplement: Supplementary file 1 [file viruses-13-02264-s001.zip › viruses-1418455-supplementary.pdf]

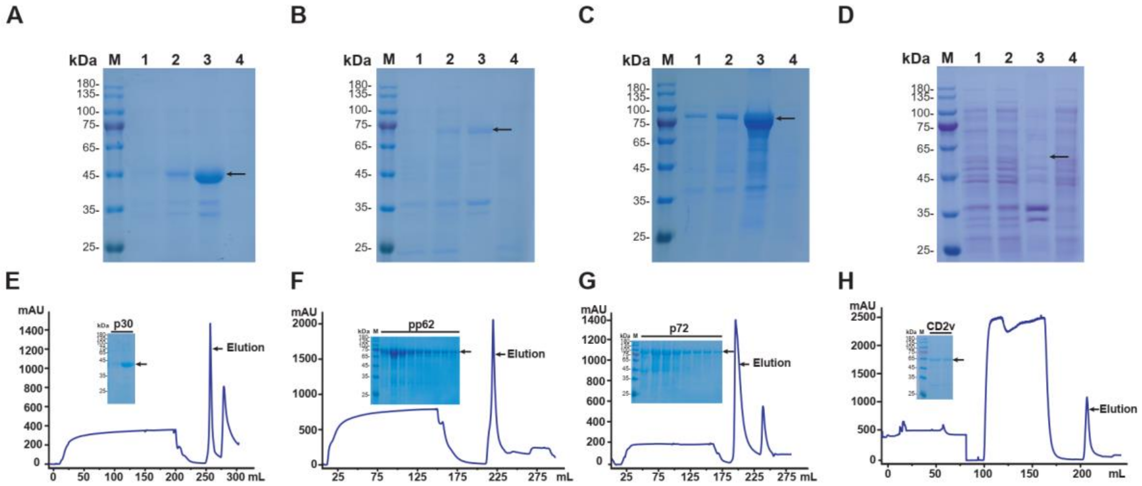


**Figure S1.** The expression and purification of ASFV proteins in *E. coli* BL21 was detected by SDS-PAGE. Related to Figure 1. SDS-PAGE data showing the expression of p30 (**A**), pp62 (**B**), p72 (**C**), and CD2v (**D**), respectively. ASFV proteins p30 (**E**), pp62 (**F**), p72 (**G**), and CD2v (**H**) expressed as inclusion bodies were purified by Ni-affinity chromatography by AKT protein purification system. M, Marker; 1, Before induction; 2, After induction; 3, Inclusion body; 4, Supernatant; Black arrow shows the location of the target protein.

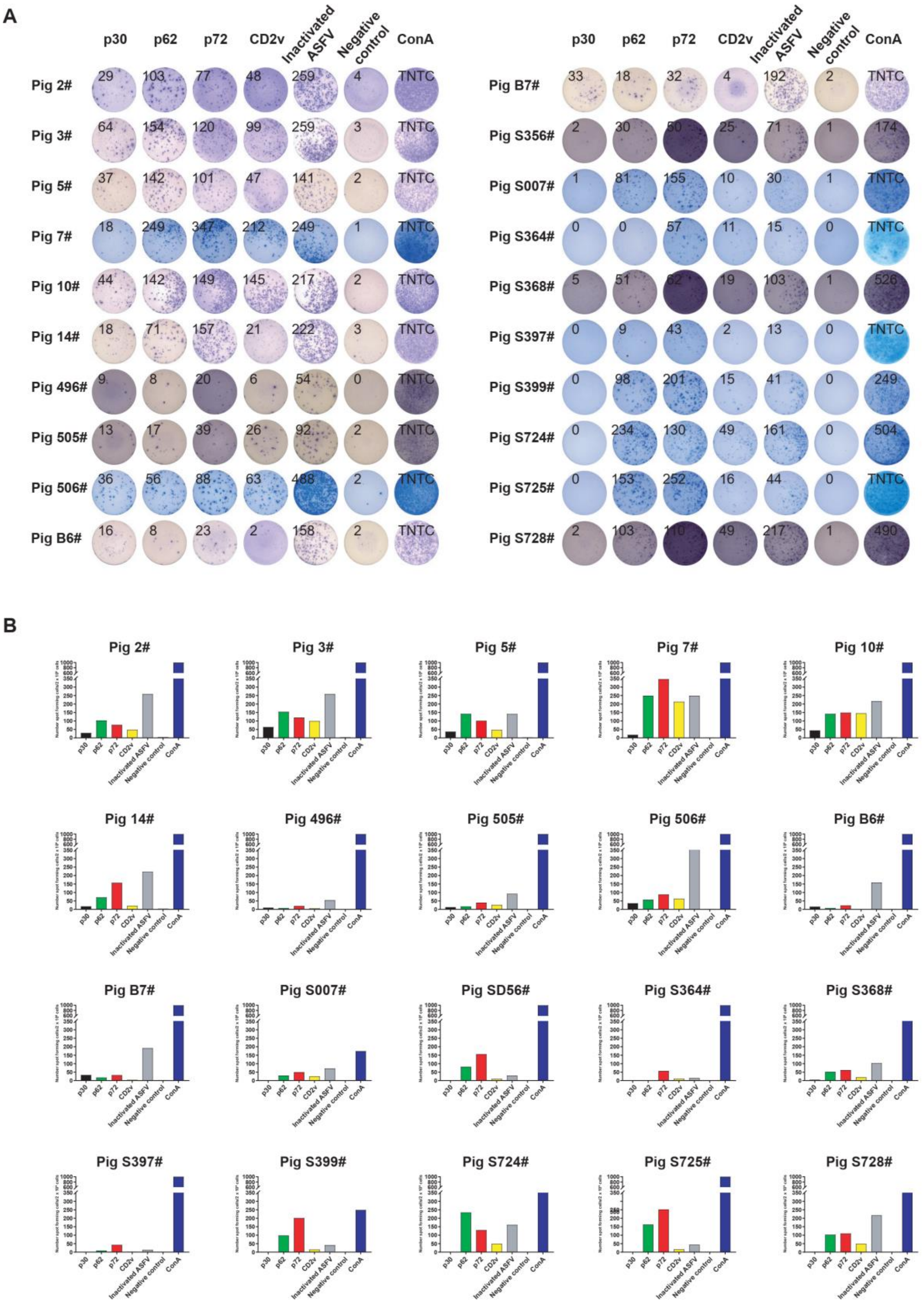


**Figure S2.** The ELISPOT assays of protein-specific T cells. Related to Figure 1. (**A**) The ELISPOT data showed immune spots formed by IFN-γ secretion after PBMC stimulation in surviving pigs. PBMCs (2×10$^5$/well) were stimulated with ASFV proteins at a final concentration of 10 μg/mL. ConA was applied as a positive stimulus. The ELSPOT data were captured by an automatic ELISPOT reader, and the number of spots was analyzed by ImmunoCapture 6.5.0 software. (**B**) Statistical analysis of the proportion of spots per surviving pig.

| Positon | P334 | SLA-1*0101 Interacting Residues | P351 | SLA-3*0301 Interacting Residues | P366 | SAL-1*1201 Interacting Residues |
|---|---|---|---|---|---|---|
| P1 | S | GLU-166 | S | TYR-7; TYR-59; GLU-63 | S | GLU-170; GLU-63 |
| P2 | L | / | R | ASN-66 | S | GLU-63; LYS-66 |
| P3 | D | TYR-59; TYR-159; TYR-7 | I | TYR-159 | Y | GLU-152 |
| P4 | E | / | S | TYR-159 | I | / |
| P5 | Y | GLU-63; GLN-67 | N | SER-24 | P | / |
| P6 | S | / | I | TYR-99 | F | / |
| P7 | S | / | K | GLU-62 | H | / |
| P8 | D | / | N | GLN-155 | Y | / |
| P9 | V | / | V | / | G | GLU-152 |
| P10 | T | ARG-155 | N | GLN-155; TRP-156 | G | THR-80 |
| P11 | T | / | K | TRP-147 | N | / |
| P12 | L | ASN-80; TYR-84 | S | / | A | LYS-146 |
| P13 | / | / | Y | ASN-77; ASN-80; TYR-84; ASP-116; TYR-118 | I | / |
| P14 | / | / | / | / | K | ASN-86 |

**Figure S3.** Each amino acid of the epitopes P334, P352, and P366 interact with amino acid in the SLA-I allele pocket. Related to Figure 3.

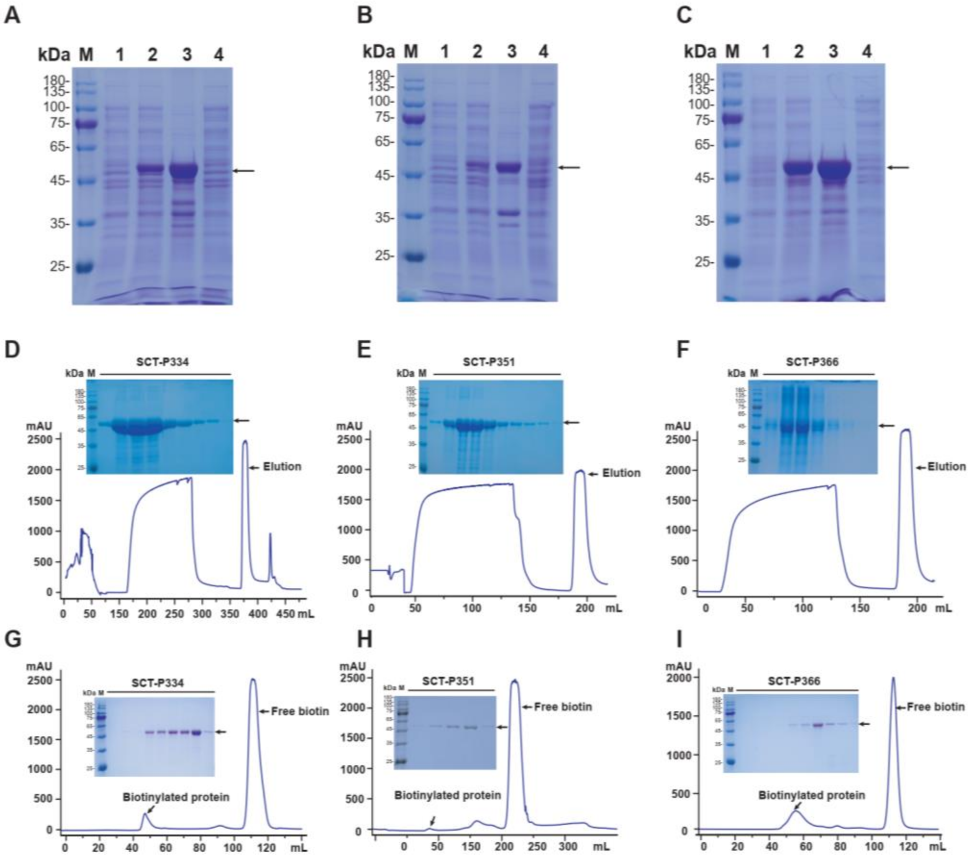


**Figure S4.** The expression and purification of tetramer protein was detected by SDS-PAGE. Related to Figure 3. (**A**, **B**, **C**) Tetra-334, Tetra-351, and Tetra-366, respectively. (**D**, **E**, **F**) SCT inclusion bodies were purified by Ni-affinity chromatography. (**G**, **H**, **I**) Biotinylation of SCT was purified by the AKT protein purification sys-tem using size exclusion chromatography. M, Marker; 1, Before induction; 2, After induction; 3, Inclusion body; 4, Supernatant; Black arrow shows the location of the target protein.

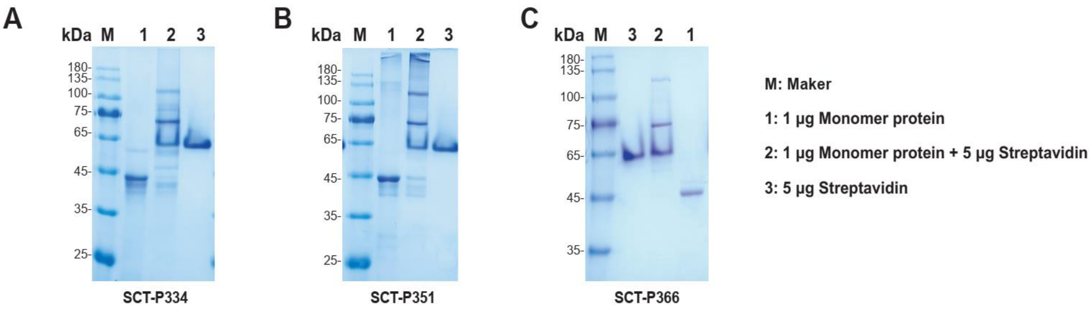


**Figure S5.** The biotinylation efficiency of the SLA-I-peptide complex was evaluated by SDS-PAGE gel-shift assays. Related to Figure 3.

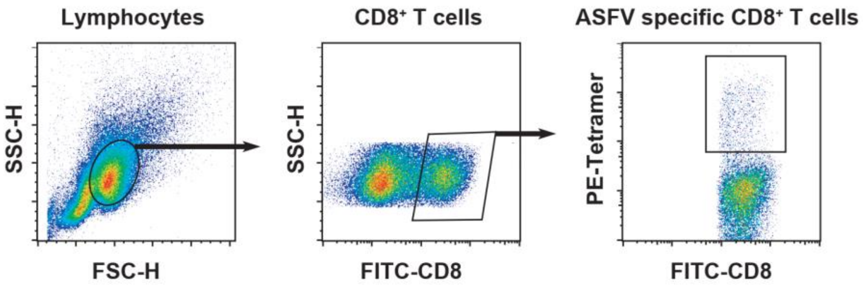


**Figure S6.** Gating strategy for PBMCs staining flow cytometry. Related to Figure 5.

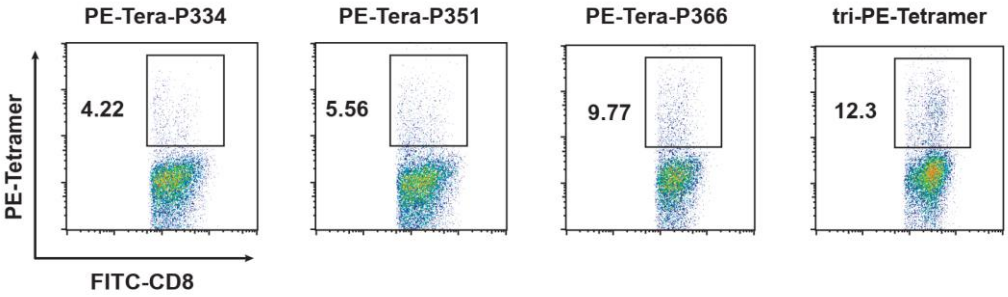


**Figure S7.** The proportion of CD8+ specific T cells of surviving Pig 10# was measured by flow cytometry assays of PBMCs stained with PE-Tera-P334, PE-Tera-P351, PE-Tera-P366 and tir-PE-Tetramer. Related to Figure 5.

**Table S1.** The general information of the surviving pigs.

| No. | Pig number | Days of age | Sampling time | Storage condition | |
|---|---|---|---|---|---|
| | | | | PBMCs | Serum |
| **1** | **Pig 2#** | **50-day-old** | 60 dpi* | $LN_2$* | -80°C |
| 2 | Pig 3# | 50-day-old | 58 dpi | $LN_2$ | -80°C |
| 3 | Pig 5# | 50-day-old | 63 dpi | $LN_2$ | -80°C |
| 4 | Pig 7# | 50-day-old | 60 dpi | $LN_2$ | -80°C |
| 5 | Pig 10# | 50-day-old | 62 dpi | $LN_2$ | -80°C |
| 6 | Pig 14# | 50-day-old | 65 dpi | $LN_2$ | -80°C |
| 7 | Pig 496# | 50-day-old | 48 dpi | $LN_2$ | -80°C |
| 8 | Pig 505# | 50-day-old | 43 dpi | $LN_2$ | -80°C |
| 9 | Pig 506# | 50-day-old | 50 dpi | $LN_2$ | -80°C |
| 10 | Pig B6# | 50-day-old | 40 dpi | $LN_2$ | -80°C |
| 11 | Pig B7# | 50-day-old | 55 dpi | $LN_2$ | -80°C |
| 12 | Pig S007# | 50-day-old | 55 dpi | $LN_2$ | -80°C |
| 13 | Pig S356# | 50-day-old | 51 dpi | $LN_2$ | -80°C |
| 14 | Pig S364# | 50-day-old | 48 dpi | $LN_2$ | -80°C |
| 15 | Pig S368# | 50-day-old | 48 dpi | $LN_2$ | -80°C |
| 16 | Pig S397# | 50-day-old | 41 dpi | $LN_2$ | -80°C |
| 17 | Pig S399# | 50-day-old | 58 dpi | $LN_2$ | -80°C |
| 18 | Pig S724# | 50-day-old | 66 dpi | $LN_2$ | -80°C |
| 19 | Pig S725# | 50-day-old | 66 dpi | $LN_2$ | -80°C |
| 20 | Pig S728# | 50-day-old | 66 dpi | $LN_2$ | -80°C |

* days post infection (dpi); liquid nitrogen ($LN_2$)
